# The Role of Histone Acetylation-/Methylation-Mediated Apoptotic Gene Regulation in Hepatocellular Carcinoma

**DOI:** 10.3390/ijms21238894

**Published:** 2020-11-24

**Authors:** Pradeep Kumar Rajan, Utibe-Abasi Udoh, Juan D. Sanabria, Moumita Banerjee, Gary Smith, Mathew Steven Schade, Jacqueline Sanabria, Komal Sodhi, Sandrine Pierre, Zijian Xie, Joseph I. Shapiro, Juan Sanabria

**Affiliations:** 1 Department of Surgery, Marshall University School of Medicine, Huntington, WV 25701, USA; rajan@marshall.edu (P.K.R.); udohu@marshall.edu (U.-A.U.); sanabriaju@marshall.edu (J.D.S.); smith2152@live.marshall.edu (G.S.); schade4@live.marshall.edu (M.S.S.); sanabriaja@marshall.edu (J.S.); sodhi@marshall.edu (K.S.); 2 Marshall Institute for Interdisciplinary Research (MIIR), Marshall University School of Medicine, Huntington, WV 25701, USA; banerjeem@marshall.edu (M.B.); pierres@marshall.edu (S.P.); xiez@marshall.edu (Z.X.); shapiroj@marshall.edu (J.I.S.)

**Keywords:** histone modification, histone acetylation, histone methylation, NAFLD, NASH, HCC

## Abstract

Epigenetics, an inheritable phenomenon, which influences the expression of gene without altering the DNA sequence, offers a new perspective on the pathogenesis of hepatocellular carcinoma (HCC). Nonalcoholic steatohepatitis (NASH) is projected to account for a significant share of HCC incidence due to the growing prevalence of various metabolic disorders. One of the major molecular mechanisms involved in epigenetic regulation, post-translational histone modification seems to coordinate various aspects of NASH which will further progress to HCC. Mounting evidence suggests that the orchestrated events of cellular and nuclear changes during apoptosis can be regulated by histone modifications. This review focuses on the current advances in the study of acetylation-/methylation-mediated histone modification in apoptosis and the implication of these epigenetic regulations in HCC. The reversibility of epigenetic alterations and the agents that can target these alterations offers novel therapeutic approaches and strategies for drug development. Further molecular mechanistic studies are required to enhance information governing these epigenetic modulators, which will facilitate the design of more effective diagnosis and treatment options.

## 1. Introduction

Epigenetic phenomena are heritable adaptive reversible changes in gene expression, or the phenotype modulated by environmental stimuli, which are not induced by changes in the DNA sequence [1]. Histone post-translational modification (PTM) represents a major contributor to epigenetic regulation of gene expression, as one aspect of an ever-growing network of epigenetic regulators [2]. In addition to histone PTMs, a long list of regulatory mechanisms of gene expression fit under this term including DNA methylation, microRNAs, long noncoding RNAs, and methyl-DNA-binding proteins. As recent evidences point out, histone modifications have a role in gene transcription, DNA repair, mitosis, meiosis, development, and in apoptosis [3,4]. The regulated nature of apoptosis makes it likely that nuclear changes experienced by apoptotic cells are mediated by epigenetic markers [5]. In the context of apoptosis, histone modifications have long been suggested to affect chromatin function and structure during cell death [6]. Studies in recent years have shown that epigenetic changes of the chromatin state are fundamental parameters of the nuclear rearrangements experienced by apoptotic cells that can be further responsible for various disease states including cancer [7,8,9].

Hepatocellular carcinoma (HCC) accounts for 90% of primary liver cancers and occurs in the background of chronic liver disease and cirrhosis of the liver [10,11]. The whole spectrum of nonalcoholic fatty liver disease (NAFLD), ranging from fatty liver to nonalcoholic steatohepatitis (NASH) to cirrhosis, is recognized as the most common cause of chronic liver disease worldwide, with a global prevalence of 25.2% [12]. The rising incidence of these metabolic abnormalities has subsequently led to a significant upsurge in NASH-related HCC [10,13]. The development of HCC is a complex process of dysregulated cellular and molecular events driven by genetic and epigenetic abnormalities [14,15,16]. The lifestyle aspects such as physical inactivity, over nutrition, metabolic disorders like insulin resistance, and weight gain are reported to influence the development and progression of NASH and HCC via epigenetic mechanisms [17,18]. The epigenetic modulation of gene expression can induce phenotypic changes, without changing the DNA sequence, which offers a new perspective on the progression of HCC [19,20]. Hence, study of epigenetics, relating to alterations in chromatin states to the cellular phenotypes in liver pathology, has become a research area of considerable interest. This review focuses on the acetylation-/methylation-mediated post-translational histone modifications that have been shown to be regulated during apoptosis and its implications in NAFLD to NASH and HCC pathogenesis.

## 2. Histone Modifications and Its Biological Importance

**Figure 1:**
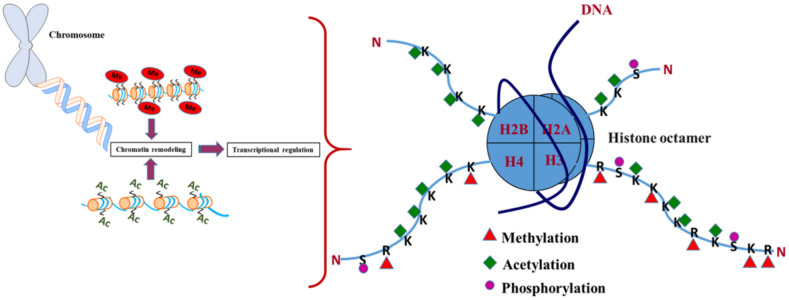
Major histone modifications associated with nucleosomal organization.

Histone modification is a covalent post-translational modification of histone proteins, which comprises of methylation, phosphorylation, acetylation, ubiquitylation, and sumoylation. These modifications can control gene expression by altering chromatin structure. Histone modifications act in diverse biological processes such as transcriptional activation/inactivation, chromosome packaging, and DNA damage/repair. Histones are small, basic nuclear proteins associated with DNA that help package DNA into chromosomes. Modification of histones affect their charge and ability to bind and position on DNA, in order to interact with other nonhistone proteins. PTMs of histones is a major regulator of chromatin compactness and accessibility [21]. These modifications affect the availability of DNA to transcription factors and RNA polymerases. For instance, methylation of histones H3 and H4 regulates the activity of origins of DNA replication [22], while on the other hand, acetylation of histones affects chromatin structure and gene transcription [23]. Each histone can undergo numerous modifications, and the combined effect of these changes serve to elicit a multitude of responses, referred to as the “histone code”. These modifications have been proposed to play a pivotal role in the regulation of gene expression (Figure 1) [24,25]. Dysregulation of PTMs and the connection between metabolism and histone modification mediates epigenetic abnormality in complex diseases including cancer, diabetes, and neurodegeneration [26,27,28,29].

### 2.1. Histone Methylation: Epigenetic Mechanism and Factors Involved

The inherited or acquired mutations in the major factors that regulate the methylation of DNA, RNA, and/or histones are highly conserved and coordinate the regulation of gene expression that are observed in developmental disorders, ageing, and cancer [30]. The attachment of methyl groups to histone proteins occurs predominantly at specific lysine or arginine residues on histones H3 and H4 [31,32]. This modification is stable when compared to acetylation, phosphorylation, and ubiquitination, which can be mainly explained by evidences based on the mechanism of methyl group turnover [33,34,35]. Methylation of histone lysine or arginine indirectly influences the recruitment and binding of different regulatory proteins to chromatin without affecting the electrostatic bond between DNA and histones [36]. The loosening or restriction of the chromatin structure due to histone methylation or demethylation results in transcriptional repression (heterochromatin) or activation (euchromatin). The factors that determine whether histone methylation at gene promoters will repress or promote gene transcription are the target amino acid site, number of bound methyl groups, and specific enzymes present.

**Figure 2:**
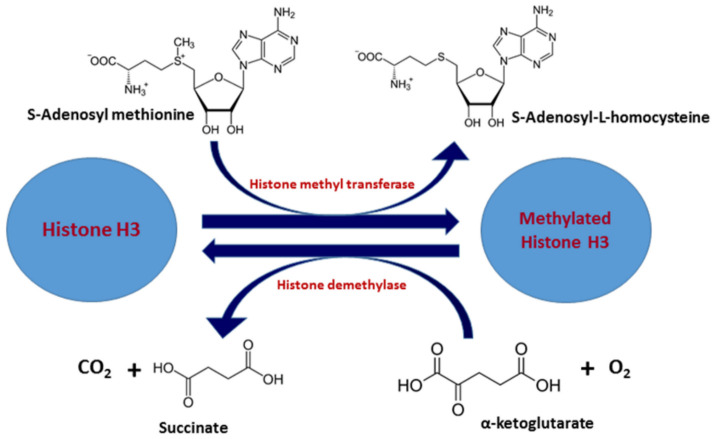
Chemical reactions involved in histone methylation reaction.

Histone methylation is mediated by histone methyltransferases (HMTs), and histone demethylation is carried out by histone demethylases (HDMs) (Zhang, 2001). Depending upon the site of methylation, there are two types of HMTs: histone arginine N-methyltransferase and histone lysine N-methyltransferase. HMTs catalyze the transfer of one to three methyl groups from S-adenosyl methionine (SAM) to the side-chain nitrogen of lysine or arginine residues (Figure 2). During the methylation reaction, SAM is converted into *S*-adenosyl homocysteine (SAH), which inhibits methyltransferase activity [37]. Thus, methyltransferases are susceptible to changes in the cellular SAM-to-SAH ratio. The role of various factors that are required for the recruitment of histone methyltransferases to various transcription sites for methylation-mediated modifications have been reported previously (Wood and Shilatifard, 2004). Methylation patterns can change under stress and disease, and increase the cellular levels of SAM, which, in turn, can repress proto-oncogenes through methylation of their promoters [38,39]. Protein arginine methyltransferases (PRMTs) methylate arginine residues on histone resulting in formation of NG-monomethylarginine, NGNG-symmetric dimethylarginine (in which both guanidino nitrogens are methylated), or NGN’G-asymmetric dimethylarginine (in which only one guanidino nitrogen receives two methyl groups) [40,41]. There are three classes of arginine methyltransferase that are conserved from yeast to human [42,43,44]. Many of the arginine methyltransferases also form homodimers, or homo-oligomers a step that is required for their catalytic activity [45]. In addition, two types of histone lysine methyltransferases have been extensively studied in chromatin biology and cause lysine specific methylation [46,47,48,49,50]. However, histone demethylases (HDMs) are “eraser” enzymes that site-specifically remove the methyl group(s) from histone lysine residuals. Based on the catalytic mechanism and sequence homology, HDMs can be further divided into classes and subfamilies [36,51,52]. The various classes and enzymes involved in histone methylation are summarized in Table 1.

### 2.2. Histone Acetylation: Epigenetic Mechanism and Factors Involved

Histone acetylation manipulates the higher order folding properties of the chromatin fiber and is associated with multitude functions including regulation of nucleosome assembly, folding and deconvolution of chromatin, heterochromatin silencing, and gene transcription [53]. Acetylation of ε-amino group of lysine residue on H3 and H4 histone tail can neutralize positive charge on histone protein and reducing its electrostatic interaction with negatively charged DNA, thus weakening the interaction of the nucleosome with the DNA backbone (Figure 1). This reduction in affinity leads to increased accessibility of the DNA to protein complexes, which causes the remodeling of the nucleosome so that the transcriptional machinery and other proteins can gain access to previously restricted sites within the DNA and further lead to increased gene expression [54]. The histone acetylation/deacetylation switches depending upon different physiological conditions and the balance between these modifications is achieved through the action of two enzyme families: histone acetyl transferases (HATs) and histone deacetylases (HDACs).

**Table 1:** Various classes/families/subtypes of enzymes involved in histone methylation and acetylation reactions.

Classification	Family/Type			Enzymes	References
HMTs	PRMTs	TYPE I		PRMT 1, PRMT 3, PRMT 4/CRM1, PRMT-6, PRMT-8	[42,43,44,55,56]
		TYPE II		PRMT 5, PRMT 9/FBXO11	[57]
		TYPE III		PRMT7	[57]
	KMTs	SET	SET I	EZH I, H3K27	[46,47]
			SET 2	NSD1-3, SETD2, SMYD2	[58,59,60,61]
			SUV39	SUV39H1, SUV39H2, G9a GLP, ESET//SETDB1 CLLL8/SETDB2	[62,63,64,65,66]
			RIZ	RIZ 1, BLIMP1/PRDM1 PFM1/CRS2	[67]
			Nongroup	SET7/9, SET8, SUV4-20H1 SUV4-20H2	[61,68]
		Seven-β-strand (7BS)		Dot1/DOT1L	[49]
HDMs	KDM1			KDM1A, KDM1B	[36]
	JMJC			KDM2-7/8	[51,52]
HATs	GNAT			KAT2A, KAT2B	[69,70]
	MYST			KAT7, KAT8, KAT5, KAT6A KAT6B	[71,72]
	p300/CBP			KAT3B	[73]
	Transcription coactivators			KAT4, KAT12	[74]
	Steroid receptor			KAT13A, KAT13B KAT13C, KAT13D	[74]
	Cytoplasmic			HAT1, HAT4	[75]
HDACs	CLASS 1			HDAC1, HDAC2 HDAC3, HDAC8	[76,77,78,79]
	CLASS II a			HDAC4, HDAC5 HDAC7, HDAC9	[80,81,82,83]
	CLASS II b			HDAC6, HDAC10	[84]
	CLASS III			Sirtuins (SIRT 1-7)	[85]
	CLASS IV			HDAC 11	[86]

**Figure 3:**
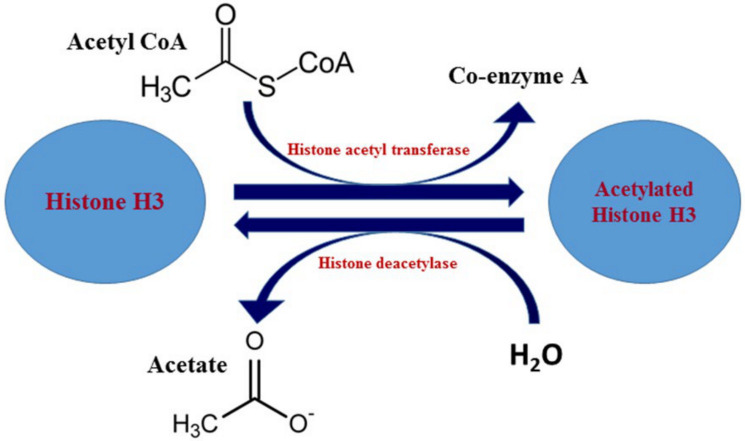
Chemical reactions involved in histone acetylation reaction.

Histone acetyltransferases (HATs) catalyze the transfer of an acetyl group from acetyl-CoA molecules to the lysine ε-amino groups on the N-terminal tails of histones (Figure 3) [87]. Acetylation of lysine residues provides unique binding surfaces for repressors and activators of transcription [88]. These enzymes are grouped into various super- and subfamilies and recently these enzymes have been identified to be important for centrosome function [69,89,90,91,92,93]. Histone deacetylases (HDACs) reverse histone acetylation and promote gene silencing. HDACs are often components of large protein complexes and are recruited to sites of DNA methylation by methyl DNA-binding proteins. HDACs remove acetyl groups from histone tail lysine residues and thereby work as repressors of gene expression [74,94,95,96]. Eighteen enzymes belonging to the HDAC superfamily have been identified and are further subdivided into various classes [76,77,79,82,97]. The various classes and enzymes involved in histone acetylation are summarized in Table 1.

## 3. Regulatory Mechanism of Apoptosis by Histone Acetylation/Methylation

The activation of a genetically controlled cell death program leading to apoptosis results in characteristic biochemical and morphological features and is essential for the morphogenesis, development, differentiation, and homeostasis of eukaryotic multicellular organisms [98]. Due to the regulated nature of apoptosis, epigenetic features are expected to have a major role in the nuclear changes associated with apoptotic cells, and the mechanistic studies are still under investigations. The major chemical modification of histone that are reported to be involved in the chromatin alterations in cells undergoing apoptosis, termed as the apoptotic histone mark, are phosphorylation of histone H2A, H2B, H3, and H4, dephosphorylation of histone H1, acetylation of histone H2B, H4, hypoacetylation of histone H4, methylation of histone H3 and H4, and de-ubiquitylation of histone H2A [8]. The epigenetic modifications in histone and DNA released during apoptosis are mainly characterized by the methylation and acetylation status of histone from perinuclear heterochromatin and hypermethylation of DNA [99]. These modifications may provide cells with specific nuclear structures, specific substrates necessary to initiate the degradation of the nucleus in apoptosis and to control chromatin condensation. As the first initial step of chromatin condensation during early apoptosis, the hyperacetylated endonuclease-hypersensitive euchromatin would be degraded, accompanied by the degradation of both the nuclear lamina and components of the intranuclear protein matrix [99]. The collapse and aggregation of heterochromatin leads to the characteristic apoptotic chromatin condensation followed by a degradation process of the heterochromatin [100]. Some studies report the separation of the core nucleosomal histones H2A, H2B, H3, and H4 from DNA, in response to apoptotic signals from a death receptor (CD95 and TNF-α) or mitochondrial apoptotic stimulus [101]. Numerous studies report the inter-relation of histone acetylation/deacetylation mechanisms and apoptosis-related pathways [102,103,104,105].

The histone deacetylase inhibitors (HDACis), increase the overall levels of acetylation on histones in cells to alter gene expression, cause cell cycle arrest, induce cellular differentiation and causes apoptosis and hence they are widely used as anticancer drugs [106]. HDACis modulate the balance between pro- and antiapoptotic proteins to induce both extrinsic and intrinsic apoptotic pathways, and acetylate nonhistone proteins that are associated with apoptosis. HDACis activate the mitochondrial apoptotic pathway by transcriptional activation of proapoptotic proteins: thioredoxin binding protein 2, BAK, Bax, Apaf-1, Bad, Bim, Bid, caspase-3, and caspase-9; and repression of antiapoptotic proteins: thioredoxin, Bcl-2, Bcl-XL, XIAP, Mcl-1, and survivin [107]. Hajji and coworkers showed that HDACis in combination with conventional chemotherapeutic drugs could be valuable in the treatment of malignancies in which Bcl-xL is overexpressed [108]. A histone deacetylase inhibitor, valproic acid enhances acetylation of histone H3 K9 and NF-κB lysine 310, which, in turn, induces NF-κB activation, reduces JNK activation, and protects the neurons from hypoxia-induced apoptosis in vitro [109].

In a study of the structural changes that occur in chromatin of apoptotic cells, Allera and coworkers described that histones became deacetylated in rat thymocytes during apoptosis induced by glucocorticoids [110]. The percentages of monoacetylated and diacetylated H4 decreased with a corresponding increase in the percentage of unmodified H4, suggesting that the bulk deacetylation could promote chromatin condensation by allowing greater DNA–histone interactions and conformational changes at the nucleosomal level. Acetylation of H4 on lysine 16 (H4-K16Ac) is a prevalent and reversible post-translational chromatin modification in eukaryotes [111]. H4-K16Ac also inhibits the ability of the adenosine triphosphate utilizing chromatin assembly and remodeling enzyme to mobilize a mononucleosome, indicating that this single histone modification modulates both higher order chromatin structure and functional interactions between a nonhistone protein and the chromatin fiber [111]. The histone acetyltransferase hMOF/SIRT1 enzymatic system regulates H4K16 acetylation, changes associated with cancer occurrence and sensitivity to topoisomerase inhibitor [112].

Expression of monomethyl histone H3 lysine 27 was reported in staurosporine (a PKC inhibitor)-induced apoptotic osteosarcoma cells [113]. Studies also report the regulation of lifespan longevity by H3K4 methyltransferase/demethylase complex [114]. Restoration of the chromatin following double-strand break (DSB) repair is driven by acetylated H3K56 suggesting a signal for the completion of repair [115]. Walter described loss of H3K4 methylation due to depletion of the methyltransferase Set1p, as the step to enhanced cell death during chronological aging and increased sensitivity to apoptosis induction. In contrast, loss of H3K79 methylation due to Dot1/DOT1L (disruptor of telomeric silencing 1, a histone methyltransferase that methylates lysine 79 located within the globular domain of histone H3) slightly affects yeast survival [116]. Therefore, aged and dying wild-type cells lose H3K4 methylation, whereas depletion of the H3K4 demethylase, Jhd2p, improves survival, indicating that loss of H3K4 methylation is an important trigger for apoptotic cell death [117]. A global histone hypoacetylation and histone H4 trimethylation at Lys-20 are reported to act as a signal for enzymes that are capable of inducing DNA fragmentation and/or chromatin condensation [99]. In leukemia cells, it was shown that inhibition of DOT1L, the sole human homolog of yeast Dot1, and H3K79 methylation increased apoptosis due to downregulation of the antiapoptotic protein BCL2L1 [118]. The ribosomal DNA silencing is reported to be affected by changes in H4K16ac, which may lead to nucleolar stress and an apoptotic response [119]. The aforementioned literature survey provides significant bridge information between the histone methylation-/acetylation-mediated epigenetic episodes that affect chromatin function and structure during apoptotic process, which can be involved in various disease conditions including cancer.

## 4. Role of Histone Acetylation-/Methylation-Mediated Apoptosis in NAFLD

**Figure 4:**
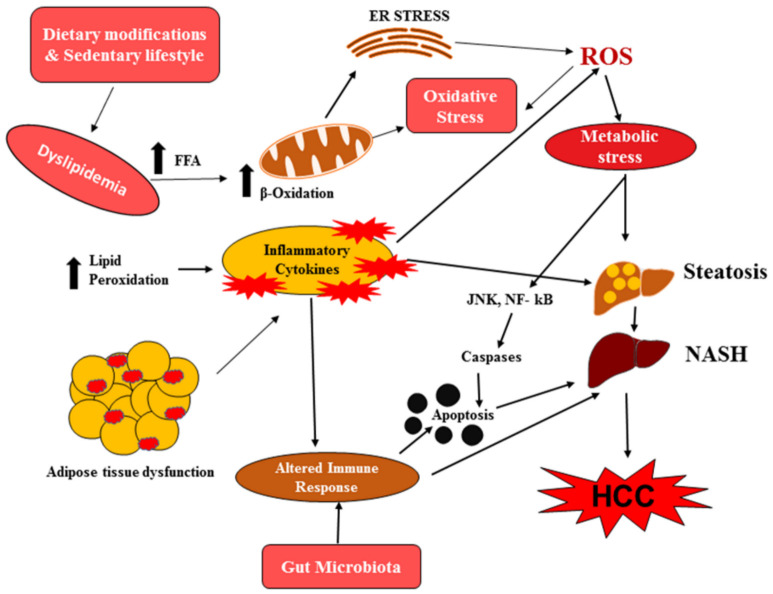
Schematic representation of metabolic and molecular changes associated with apoptotic pathway in nonalcoholic fatty liver disease (NAFLD) and hepatocellular carcinoma (HCC).

Epigenetic factors are involved in the regulation of hepatic lipid metabolism, insulin resistance, endoplasmic reticulum stress, mitochondrial damage, oxidative stress response, and inflammation, all of which have been implicated in the development and progression of NAFLD [120,121,122,123] and will further trigger carcinogenesis of hepatocytes and facilitate the progression to HCC [124,125]. Emerging studies suggests that increased hepatocyte apoptosis is a crucial mechanism that contributes to the activation and progression of NASH [126]. Activation of caspases, Bcl-2 family proteins, and c-Jun N-terminal kinase-induced hepatocyte apoptosis plays a role in the liver inflammation and fibrogenesis during NASH [127]. Monounsaturated and saturated fatty acids have been reported to induce the activation of endoplasmic reticulum (ER) stress-associated c-Jun N-terminal kinase (JNK), which in turn promotes hepatocyte apoptosis by modifying the expressions of proapoptotic members of the Bcl-2 family [128]. ER stress can also drive lipogenesis and steatohepatitis via caspase-2 activation of site 1 protease and further abnormalities in glucose and lipid metabolism, production of reactive oxygen species, oxidative stress, as well as microbiota accelerate the processes of hepatocyte apoptosis in NASH [129,130]. Apoptotic hepatocytes also stimulate immune cells and hepatic stellate cells and leads to the progression of fibrosis in the liver by the production of inflammasomes and cytokines [127]. Cytokeratin-18 (CK-18) fragments generated by caspase 3 activation are a major independent predictor of NASH [130]. Dead hepatocytes are engulfed by macrophages, leading to the release of proinflammatory signals that activate stellate cells, ultimately resulting in fibrosis (Figure 4) [131]. The epigenetic alterations that are regulated by the environmental factors such as nutrition and diet, drugs, and stress are mainly DNA methylation, histone modifications, and microRNAs [17,132,133]. As alterations in epigenetic factors could distinguish between NAFLD/NASH stages, a better understanding of the molecular mechanisms will help to develop novel approaches for reliable biomarkers and effective treatments. Altered expression and activity of various histone acetylation and methylation modifying enzymes have been reported to influence gene expression in NAFLD/NASH, leading to altered hepatic metabolism, apoptosis, and cellular transformation including HCC progression [125]. p300, a member of the HAT family, is a key transcriptional controller that is involved in the NF-kB-dependent inflammatory pathways [134]. It has been reported that p300 and serine/threonine kinase salt-inducible kinase 2 (SIK2) are major upstream regulators of carbohydrate-responsive element-binding protein (ChREBP) activity, which acts as a transcriptional activator of lipogenic and glycolytic genes and therefore, specific SIK2 activators and p300 inhibitors may be useful in pharmaceutical intervention of NAFLD/NASH [135]. The recruitment of NF-κB p65 to gene promoters and subsequent expression of NF-κB induced inflammatory cytokines are reported to be affected by methyltransferase SET7/9, which targets lysine residue 4 of histone H3 (H3K4) [136].

Histone acetylation of sterol 12α-hydroxylase (CYP8B1) gene promoter induced by retinoic acid-related orphan receptor α (RORα) regulates bile acid synthesis and cholesterol levels and has been implicated in dyslipidemia-associated inflammatory changes in NAFLD/NASH [137]. An increase in histone H3 lysine 9 and 18 acetylation at TNFα and CCL2 have been reported in obesity and fatty liver [138]. Studies have shown that the aberrant histone H3K4 and H3K9 trimethylation in PPARα and lipid catabolism-related genes leads to hepatic steatosis and disease progression [139]. The epigenetic modification of SIRT1 and SIRT3 plays a major role in the regulation of glucose homeostasis, antihyperlipidemic activity, insulin sensitivity, oxidative stress, anti-inflammatory activity, and antiaging activity. The deacetylation of SIRT1, has been reported as a regulator of various proteins that are involved in the pathophysiology of NAFLD/NASH [140]. Hepatic mitochondrial protein hyperacetylation and decreased SIRT3 expression have also been reported in high fat diet [141]. The NAD-dependent sirtuins (class III HDAC, SIRT), which target both histones and nonhistone proteins, mediate adaptive responses to metabolic stress, and regulate adipogenesis and insulin secretion [141,142]. As discussed previously, histone modification-mediated altered metabolic and molecular events will further lead to the sequential activation of apoptotic signaling and progression of NAFLD to NASH through hepatocyte apoptosis and that ultimately will pave the way to HCC. Hence, understandings of this epigenetic mechanism underlying NAFLD may provide lead for the development of future diagnostic targets for HCC and associated comorbidities.

## 5. Role of Histone Acetylation-/Methylation-Mediated Apoptosis in HCC

Lipotoxicity, oxidative stress, inhibition of hepatic autophagy, proinflammatory cytokines, Toll-like receptor signaling, altered bile metabolism, and compromised T-cell function promote the development of NASH-associated HCC [143]. As discussed in the previous section, various metabolic, inflammatory, and molecular alterations associated with NASH leads to hepatocyte apoptosis, and this excessive apoptosis may induce compensatory proliferation which, in turn, can promote tumorigenesis by exacerbating inflammation and oxidative stress in HCC [144]. The progression of NASH to malignancy is a multifaceted process that involves dysregulation of multiple cellular and molecular pathways driven by genetic and epigenetic alterations that affect apoptosis and enable tumor nest development. The major inflammatory cytokines such as TNF-α and interleukin-6 induced NF-κB pathway and the downstream signaling of c-Jun N terminal kinase (JNK), which plays a pivotal role in inflammation-associated HCC [145]. NF-κB activation can cause the induction of several antiapoptotic molecules including, Bcl-2, Bcl-X, cFLIP, TRAF1, TRAF2, and GADD45β, and activation of prosurvival and proproliferative pathway mediators such as p38 MAPK kinase (mitogen-activated protein kinase) [146,147,148]. The proapoptotic factors such as Bax or Bcl-XS are downregulated in HCC with dysfunction in the p53 pathway [149]. Survivin, another member of the family of inhibitor of apoptosis proteins, which is found to be upregulated in HCC, not only inhibit apoptosis but also promote cell proliferation and metastasis [150,151]. The alteration of the immune cell composition and impairment of immune-mediated clearance of damaged hepatocytes along with dysregulation of lipid metabolism in NAFLD/NASH induces selective ablation of intrahepatic CD4^+^ cells, which impairs mitochondrial function and generates oxidative stress resulting in impaired immune surveillance [152,153].

Emerging evidence shows mutations and changes in expression of epigenetic modifiers are common events in HCC, leading to an aggressive gene expression profile and poor clinical prognosis [154]. Histone methylation is tightly connected to apoptosis by chromatin rearrangement and to regulation of metastasis and proliferation-related genes. Enhancer of zeste homologue 2 (EZH2), a histone-lysine N-methyltransferase enzyme, mediates gene silencing via H3K27me3, and it is frequently overexpressed in HCC. In addition, EZH2 is also involved in DNA damage associated with cell cycle arrest and apoptosis in cells from HCC [155]. Malignancy progression to metastasis is associated with various methyltransferases and demethylases (methylate histone H3-K4 and K36), which are responsible for conformational changes affecting the balance and distribution of euchromatin and heterochromatin, leading to the upregulation of mesenchymal–epithelial transition (MET)-related genes [24]. SETDB1 is a methyltransferase that targets histone H3K9 methylation to repress gene expression [156]. SETDB1 knockdown in HCC cell lines exhibited downregulation of T-lymphoma invasion and metastasis gene (Tiam1), reducing cancer migration and suggesting the positive correlation between SETDB1 and Tiam1 in HCC [157].

Euchromatic histone lysine methyltransferase 2 (G9a, EHMT2) and suppressor of variegation 3-9 homolog 1 (SUV39H1) are mainly involved in the methylation of histone H3K9 to induce the formation of heterochromatin, and G9a upregulation was significantly associated with malignant clinicopathological features of HCC [158]. However, knockdown studies of G9a, showed suppression of HCC cell metastasis and proliferation via the induction of retinoic acid receptor responder protein 3 (RARRES3) [159]. The loss of histone H4K20 trimethylation and deacetylation of H4K16 during liver carcinogenesis has been studied as a prominent alteration in cell death pathway [160]. KDM5C and JARID1B are histone demethylases in the family of JmjC domain-containing proteins that mainly demethylate histone H3K4 to suppress gene expression via the formation of heterochromatin and are abundantly expressed in HCC [161,162]. Demethylation of histone H3K9 and K27 leads to the formation of open chromatin structure and reported to play role in HCC cell proliferation and migration. Studies have shown that the knockdown of KDM4B (a H3K9 demethylase) induced HCC growth and metastasis via reduction in miR-615-5p expression and increased RAB24 expression [163], whereas low KDM6B (a H3K27 demethylase) levels in response to miR-941 regulation reduced HCC cell proliferation, migration, and invasion in both in vitro and in vivo [164].

A well-known histone acetyltransferase, P300/CBP-associated factor (PCAF), has been reported as an HCC repressor, which promote cell apoptosis and inhibits tumor growth, through acetylation of histone H4 [165]. Several lines of evidences suggest the dysregulation of HDACs, which are responsible for inappropriate transcriptional activation in various cancers including HCC [166,167]. HDAC1 and HDAC2 have also been found to be upregulated in HCC and this dysregulation contributes to HCC pathogenesis by modulating expression of genes involved in apoptosis, cell cycle and, lipid metabolism [168,169]. The upregulation of HDAC1 and HDAC2 suppresses the expression of a key metabolic enzyme in glucose metabolism, fructose-1, 6-bisphosphatase (FBP1), with concomitant increase in lactate production in liver cancer cells, and restoration of FBP1 expression via inhibition of HDAC1/2. The former inhibition was found to suppress cell proliferation and induce cell death [94,170]. Loss of SIRT6 prompted cells glycolytic path towards lactate production, even under aerobic conditions suggesting that the lack of SIRT6 might provide a growth benefit for tumor cells [171]. Hence, the remarkable role of acetylation-/methylation-mediated apoptosis in HCC have stimulated several researches focused on potential epigenetic modifiers. The major histone acetylation-/methylation-mediated epigenetic modifications involved in NAFLD and HCC are summarized in Table 2.

### Significance of Histone Acetylation/Methylation Ratio in HCC

The role of various residue specific histone modifications and their subsequent effect on active transcription and gene repression are studied recently to unravel the importance of complex epigenetic modification in various cancers including HCC. Most established specific methylation and acetylation residues in HCC include trimethylation of lysine 4 in histone 3 (H3K4me3), acetylation of histone 3 at lysine placed on position 18 (H3K18Ac), mono- and trimethylation at H3K27, acetylation of lysine 9 on histone 3 (H3K9ac), acetylation of lysine 8 on histone 4 (H4K8ac), methylation of histone H3 at lysine 4 (H3K4) and lysine 36 (H3K36), and methylation of histone H3 at lysine 9 (H3K9) and lysine 27 (H3K27) positions and histone H4 at lysine 20 (H4K20) [172,173,174,175,176,177,178,179,180,181]. The transition of methylation and acetylation status at various residues in the histone are currently under investigation in relation to HCC progression. Zhang team studied the role of H3K9 methylation/acetylation ratio that contributes to silencing RIZ1 in HCC [67]. Furthermore, HCC samples showed that HCC tissues have significantly higher H3K27ac and H3K27me3 scores compared with the background liver. In addition, an aggressive HCC phenotype has been associated with acetylation and trimethylation of H3K27, which, in turn, is associated with p53 abnormalities [182]. Disruption of deacetylation by the ablation of HDAC3 had also been implicated in the reduction in trimethylation of H3K9 (H3K9me3) playing a pivotal role in the double-strand break (DSB) repair and the accumulation of damaged DNA. Finally, deacetylation of H3K9ac mediated by HDAC3 is critical for H3K9 methylation [183]. The hyperacetylated H3K9 (H3K9ac) serves as a transcriptional activator of various carcinoma-related genes and activates multiple signaling pathways to promote tumorigenesis.

## 6. Epigenetic Therapeutic Implications for HCC

Epigenetic therapy is the use of drugs or other epigenome-influencing techniques to treat many diseases, including cancer, heart disease, diabetes, and mental illnesses that are regulated by epigenetic mechanisms. Owing to the reversible nature of modifications and its broad involvement in a wide range of diseases, epigenetics had received great attention for the discovery of novel therapeutic agents. Epigenetic changes can precede disease pathology and thus they can serve as risk factors, i.e., markers for disease progression or response to treatment [184]. Studies toward understanding the role of external factors in HCC epigenetic alterations could explore the complex multifactorial origin, giving light to promising strategies for prevention and treatment [185,186,187]. Histone deacetylase inhibitors (HDACi) constitute a relatively new class of chemotherapeutic drugs and are now approved by the FDA as anticancer agents. Specific SIK2 activators and p300 inhibitors can be useful in pharmaceutical intervention of NAFLD/NASH [123,135]. Panobinostat (LBH589) is a pan-HDAC-inhibitor with high efficacy in several preclinical models of cancer [188], and treatment of panobinostat combined with sorafenib demonstrated higher preclinical efficacy in HCC [189]. The pharmacologic activation of SIRT1 with phytochemicals like resveratrol also offer a potential therapeutic strategy for NAFLD management [123]. Inhibition of histone demethylase (JMJD2B)–PPARγ2 signaling is representing a potential therapeutic strategy against NAFLD [129]. Studies demonstrated that epigenetic reconditioning using the demethylating compound 5-azacytidine (5-AZA) shows therapeutic significance for liver cancer and is potentially attractive for the treatment of solid tumors [190].

There are some emerging epigenetic drugs that are under clinical trials in the management of HCC [191]. CUDC-101 is a multitargeted agent designed to inhibit HDAC. The Phase 1b open-label expansion study of CUDC-101 (NCT01171924) investigates the safety and tolerability of the drug in patients with tumors including HCC. Another drug that target HDAC, Belinostat, in the Phase I/II trial (NCT00321594), explores the side effects and best dose of the drug in treating patients with HCC that cannot be removed by surgery. Vorinostat (Phase 1) (NCT01075113) and 4SC-201 (resminostat—Phase 2) (NCT00943449) are HDACis that are under clinical trials in combination with sorafenib for the treatment of hepatocellular carcinoma in patients refractory to sorafenib monotherapy. The Phase 2 open-label randomized study of SGI-110 (NCT01752933), which targets DNMT, investigates the effect of subcutaneously administered drug in the treatment of advanced hepatocellular carcinoma (HCC) patients who failed prior treatment with sorafenib. Thus, the opportunity provided by better understanding and exploitation of epigenetic mechanisms operating in HCC will encourage future advances in new clinical interventions.

## 7. Conclusions and Future Perspectives

**Figure 5:**
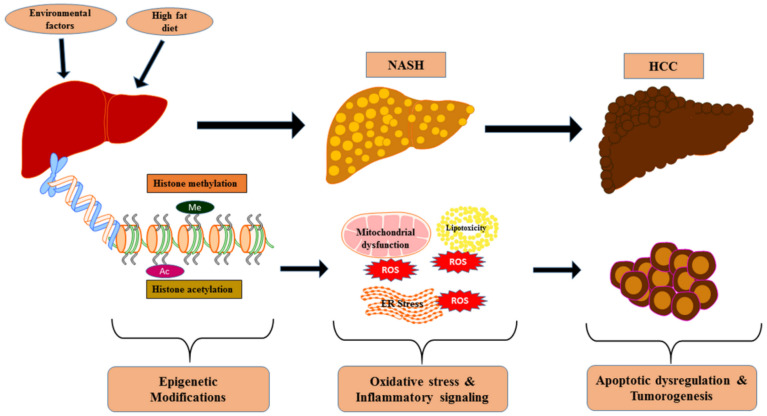
The coordinated epigenetics-mediated cellular events allied to the progression of nonalcoholic steatohepatitis (NASH) and associated HCC.

HCC is a complex disease where genetic, epigenetic, and environmental factors combine to define the development of malignancy and its progression. The coordinated epigenetics-mediated cellular events allied to the progression of HCC, discussed in this review, is schematically represented in Figure 5. Knowledge of the fundamental epigenetic mechanisms governing gene expression and cellular phenotype in NAFLD to NASH and HCC will help to further elucidate the modulation of the histone modification-mediated apoptotic pathways. These studies have laid a solid foundation for additional research to establish novel insights into epigenetic biomarkers and clinical interventions. Epigenetic profiling may have predictive or prognostic value, and epigenetic biomarkers can be used to complement current strategies for diagnosis and prediction of drug responses. However, dynamism coupled with the complexity of epigenetic mechanisms presents significant challenges and necessitates the requirement of future detailed studies by considering various cellular environments and their targets of actions at distant biological sites.

## Figures and Tables

**Table 2:** Histone acetylation-/methylation-mediated epigenetic changes in nonalcoholic fatty liver disease (NAFLD) and hepatocellular carcinoma (HCC) that leads to apoptotic pathway regulation.

Histone Modifications	Biological Effects in NAFLD/HCC	References
HAT activity of p300/SIK2	Transcriptional activation of lipogenic and glycolytic genes	[135]
HMT activity of SET7/9 on H3K4	NF-κB-induced inflammation	[136]
Histone acetylation of Sterol 12α-hydroxylase (CYP8B1) by RORα	Dyslipidemia-associated inflammatory changes and regulation of bile acid synthesis and cholesterol levels	[137]
H3K9 and H3K18 acetylation of TNF-α and CCL2	Obesity and fatty liver	[138]
H3K4 and H3K9 trimethylation in PPARα and lipid catabolism-related genes	Hepatic steatosis and NASH progression	[139]
Sirtuins	Mediate adaptive responses to metabolic stress and regulate adipogenesis and insulin secretion in NAFLD	[140,141,142]
H3K27 trimethylation by EZH2	Cell cycle arrest and apoptosis in HCC	[155]
Methylation/demethylation of histone H3-K4 and K36 of mesenchymal-epithelial transition (MET)-related genes	Malignancy progression to metastasis in HCC	[24]
SETDB1-mediated histone H3K9 methylation	Downregulation of T-lymphoma invasion and metastasis gene (Tiam1)	[156,157]
H3K9 methylation by G9a, EHMT2 and SUV39H1	Malignant clinicopathological features of HCC	[158,159]
Loss of histone H4K20 trimethylation and deacetylation of H4K16	Alteration in cell death pathway	[160]
Demethylation of H3K4 by KDM5c and JARID1B	Suppression of gene expression in HCC	[161,162]
Demethylation of histone H3K9 (KDM4B) and K27 (KDM6B)	HCC cell proliferation and migration	[163,164]
Histone H4 acetylation by P300/CBP-associated factor (PCAF)	Promote cell apoptosis and inhibits tumor growth	[165]
Activity of HDAC1 and HDAC2 on metabolic enzymes	Regulate cell proliferation and cell death in HCC pathogenesis	[94,166,167,168,169,170]

## Abbreviations

HATs	Histone acetyltransferases
HCC	Hepatocellular carcinoma
HDACis	Histone deacetylase inhibitors
HDACs	Histone deacetylases
HDMs	Histone demethylases
HMTs	Histone methyltransferases
NAFLD	Nonalcoholic fatty liver disease
NASH	Nonalcoholic steatohepatitis
PRMTs	Protein arginine methyltransferases
PTM	Post-translational modification
